# Clustering of *Pseudomonas aeruginosa *transcriptomes from planktonic cultures, developing and mature biofilms reveals distinct expression profiles

**DOI:** 10.1186/1471-2164-7-162

**Published:** 2006-06-26

**Authors:** Richard D Waite, Alberto Paccanaro, Anastasia Papakonstantinopoulou, Jacob M Hurst, Mansoor Saqi, Eddie Littler, Michael A Curtis

**Affiliations:** 1 MRC Molecular Pathogenesis Research Unit, Centre for Infectious Disease, Institute of Cell and Molecular Science, Barts and the London, Queen Mary School of Medicine and Dentistry, 4 Newark Street, London, E1 2AT, UK; 2 Department of Computer Science, Royal Holloway, University of London Egham, TW20 0EX, UK; 3 Medivir UK Ltd, Peterhouse Technology Park, 100 Fulbourn Road, Cambridge CB1 9PT, UK

## Abstract

**Background:**

*Pseudomonas aeruginosa *is a genetically complex bacterium which can adopt and switch between a free-living or biofilm lifestyle, a versatility that enables it to thrive in many different environments and contributes to its success as a human pathogen.

**Results:**

Transcriptomes derived from growth states relevant to the lifestyle of *P. aeruginosa *were clustered using three different methods (K-means, K-means spectral and hierarchical clustering). The culture conditions used for this study were; biofilms incubated for 8, 14, 24 and 48 hrs, and planktonic culture (logarithmic and stationary phase). This cluster analysis revealed the existence and provided a clear illustration of distinct expression profiles present in the dataset. Moreover, it gave an insight into which genes are up-regulated in planktonic, developing biofilm and confluent biofilm states. In addition, this analysis confirmed the contribution of quorum sensing (QS) and RpoS regulated genes to the biofilm mode of growth, and enabled the identification of a 60.69 Kbp region of the genome associated with stationary phase growth (stationary phase planktonic culture and confluent biofilms).

**Conclusion:**

This is the first study to use clustering to separate a large *P. aeruginosa *microarray dataset consisting of transcriptomes obtained from diverse conditions relevant to its growth, into different expression profiles. These distinct expression profiles not only reveal novel aspects of *P. aeruginosa *gene expression but also provide a growth specific transcriptomic reference dataset for the research community.

## Background

*Pseudomonas aeruginosa *is an opportunistic pathogen which causes a variety of infections including bacteremia in burns patients, urinary tract catheter infections and chronic colonisation of the lungs of Cystic Fibrosis patients [1]. This bacterium is a common nosocomial contaminant and its persistence as a major cause of human disease is linked to its intrinsic resistance to many antibiotics. In addition to this natural resistance, in many infections *P. aeruginosa *survives as a biofilm – adhered communities which themselves are more resistant to antibiotic therapy than free-living or planktonic organisms [2,3]. Given the decreased therapeutic options for this organism, there is a clear need for novel approaches to treat or prevent *P. aeruginosa *infection, and the key to this is to understand more about its biology. A greater knowledge of the genetics of growth of *P. aeruginosa *will help unravel the molecular basis of infection and in particular the mechanics of survival in the biofilm mode of growth.

The success of *P. aeruginosa *as a human pathogen, and its ability to thrive in diverse environmental habitats, is thought to be due to its large and complex genome (6.3 million base-pairs, 5570 open reading frames) which includes a variety of virulence factors and also its ability to alternate between a free-living and biofilm state [4]. In order to understand this flexibility we have used whole-genome microarrays to obtain transcriptomes from six conditions relevant to the growth of this organism. These conditions were two planktonic phases (logarithmic and stationary) and multiple biofilm time points (8, 14, 24 and 48 hrs). Using confocal microscopy we have previously characterised the static biofilm model used for this study, and found that confluent biofilms develop in a sequential manner from microcolonies [5]. In a preliminary analysis of this transcriptomic dataset, we found major differences in gene expression when comparing actively growing growth states with stationary phase growth states; 19.4% of the PAO1 genome was differentially expressed between log phase and stationary phase planktonic cultures and >15.5% of the PAO1 genome was differentially expressed when developing biofilms (8 hrs) was compared to confluent biofilm time-points (14, 24 and 48 hrs) [5]. In contrast we determined that developing and confluent biofilm transcriptomes were related to those of logarithmic and stationary phase planktonic cultures, respectively and that gene expression was conserved between confluent biofilms [5]. We also identified a unique set of 20 and 26 novel genes that were ≥ 2.5 fold up-regulated (*P *< 0.05) in developing biofilms and confluent biofilms respectively, when compared to all other conditions.

Although our previous analysis gave information regarding the relatedness of these different *P. aeruginosa *growth states, in this study we provide a more comprehensive analysis of this data using clustering. We performed such analysis using three different clustering methods: K-means, K-means spectral and hierarchical clustering. This analysis provides an overview of *P. aeruginosa *gene expression in conditions relevant to its lifestyle and allowed us to reveal the existence of distinct expression profiles in the transcriptomic data from these six different conditions. We were then able to observe gene expression that is unique to single conditions, or common to two or more culture conditions.

## Results and discussion

### Growth conditions used for microarray analysis

The biofilm microarray data used in this study was derived from static biofilms grown on nitrocellulose filters placed on 20% LB agar [5]. Reduced strength LB (20%) was used in order to reflect a low nutrient environment which would be encountered *in vivo*. Visual monitoring using strain PAO1 tagged with the green fluorescent protein (GFP) and confocal scanning laser microscope (CSLM) imaging demonstrated that after filters were inoculated with single *P. aeruginosa *cells, large microcolonies had formed after 8 hours which then further developed into confluent biofilms after 14 hrs [5]. No further change in biofilm architecture was observed after 24 and 48 hrs incubation. Microarray analysis using *P. aeruginosa *Affymetrix GeneChip arrays was carried out using RNA derived from four biofilm time points; 8, 14, 24, and 48 hours [5].

The planktonic culture microarray data used in this study was derived from logarithmic and stationary phase planktonic cultures grown in 250 ml Erlenmeyer flasks. Briefly, 100 ml LB broth (20%) was inoculated with 1 ml PAO1 overnight LB culture (diluted 10 fold), and the resulting cultures were grown at 37°C with agitation (200 rpm). Microarray analysis was carried out on RNA extracted after 4 hours (OD_600 _0.1) (Logarithmic phase – LP) and 24 hours growth (OD_600 _0.3) (Stationary phase – SP).

### Transcriptome analysis: clustering of microarray data reveals different expression profiles

On analysis of the microarray data, 1429 genes (26% of genome) [see Additional file 2 – Supplementary Table S1] were found to show low expression (under 50 signal units for all three replicates) for all six different conditions. The most abundant functional classes in this list were: 'transport of small molecules', 'hypothetical, unclassified, unknown', 'putative enzymes' and 'transcriptional regulators'. This list contained two characterised multidrug efflux resistance systems (MexC-MexD-OprJ and MexE-MexF-OprN) and a putative multidrug efflux resistance system (PA3521-PA3523). Also, in this group was the gene encoding the β-lactamase precursor (*ampC*) and *katB*, one of the four genes encoding possible catalases in the *P. aeruginosa *genome. Both, *ampC *and *katB *have been shown previously to be induced in biofilms by the presence of sub-inhibitory doses of imipenem [6,7] and by hydrogen peroxide [7], respectively.

**Figure 1:**
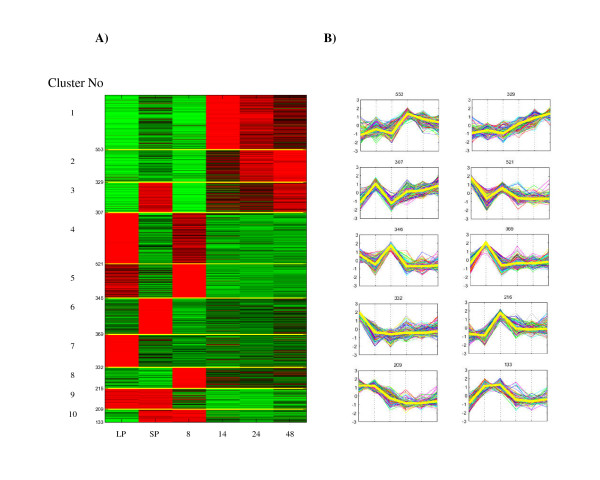
**Results of K-means clustering of the expression data (K = 10)**. The three replicates for each of the six conditions were averaged, and then normalized to zero mean and unit variance. A) The Eisen diagram [56] of the expression profiles grouped according to the results of the clustering. Red denotes that the value observed is above the mean of the observation across the dataset. The y-axis shows the number of genes in each cluster. The labels on the X-axis denote the different expression conditions: LP, LP planktonic culture; SP, SP planktonic culture; 8, 14, 24 and 48, biofilm time points. B) The plot of the profiles in each cluster. The X-axis shows the conditions in the same order as A). The thick yellow lines represent the learned cluster centres.

We began the transcriptome analysis by filtering out these 1429 genes showing very low expression for all six different conditions. A 1-way ANOVA (assuming unequal variances) was then performed which removed a further 805 genes that did not vary significantly between different conditions [see Additional file 2 – Supplementary Table S2]. As we are interested in the behaviour of genes between the six conditions and not within the 6 conditions, for the remaining 3315 genes we averaged the values of the three replicates for each of the six conditions, and then normalized these averaged profiles to zero mean and unit variance. We used the correlation coefficient as distance measure between gene profiles, and we clustered them using K-means clustering [8], K-means spectral clustering [9,10] and hierarchical clustering [8]. The first two methods were applied using a value of K (i.e. number of clusters) of 4, 7, 8, 9, 10, 12, 13, 15, 16 and 19. For the hierarchical clustering we tried the complete, centroid and ward linkage metrics for building the hierarchical tree and different values of the threshold for cutting it. An important finding is that for equivalent settings of the parameters of these different algorithms we obtained broadly the same groupings of the genes [see Additional file 1 – Supplementary Figure S1]. This revealed the existence of distinct expression profiles in the transcriptomic data from these six conditions. In the following we shall discuss the results obtained using K-means clustering with K = 10 (Figure 1a and 1b). These expression profiles are defined in Table 1.

### Confirmation of microarray transcription profiles by quantitative reverse transcriptase PCR (qRT-PCR)

**Figure 2:**
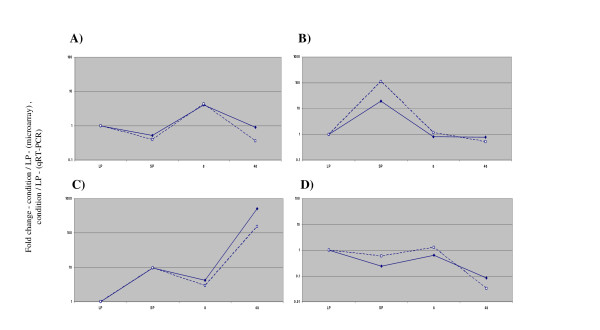
**Comparison of microarray and quantitative reverse transcriptase PCR (qRT-PCR) transcription profiles**. A) PA0020 (array cluster 8), B) *fliE *(array cluster 6), C) PA2184 – (array cluster 1), D) PA5555 – (array cluster 4). X-axis labels (expression conditions) – LP, LP planktonic culture; SP, SP planktonic culture; 8, 8 hr biofilm; 48, 48 hr biofilm. Microarray results – unbroken line, qRT-PCR results broken line. For both microarray (average expression value) and qRT-PCR (expression value) results, the value for each condition (SP, 8, and 48) was divided by the value obtained for LP.

In order to verify the results obtained using the *P. aeruginosa *GeneChip microarrays (Affymetrix), genes from four different clusters (PA0020, cluster 8; *fliE*, cluster 6; PA2184, cluster 1; PA5555, cluster 4) representing different transcriptional profiles were examined by qRT-PCR. This was performed on RNA samples taken from 4 conditions (LP planktonic culture, SP planktonic culture, 8 hr biofilm, and 48 hr biofilm) and the results are displayed relative to expression under LP planktonic culture conditions (Figure 2). The gene expression profiles were consistent with those obtained from the microarray profiling experiments. Therefore, data from qRT-PCR provides independent verification of the microarray results.

### Key functional classes in each cluster

**Figure 3:**
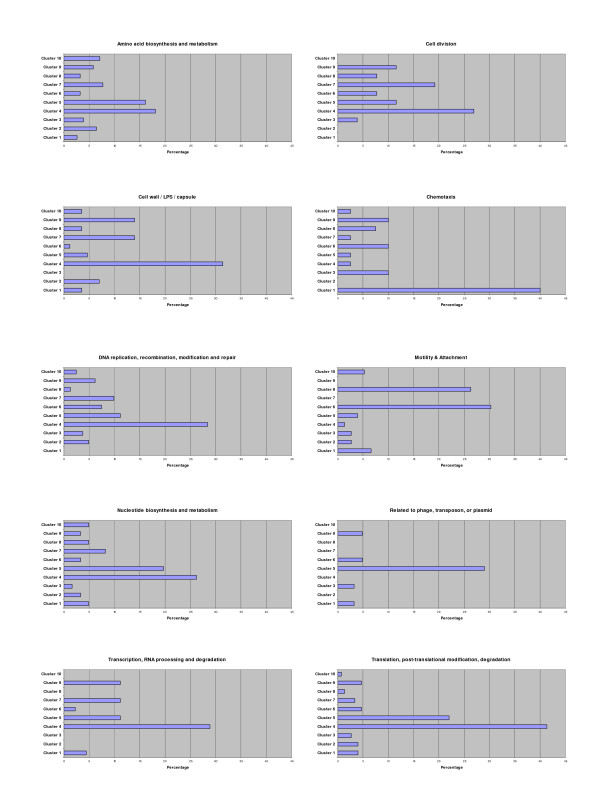
**Distribution of ten functional classes throughout the clusters created by K-means clustering (K = 10)**. Percentages were obtained by dividing the number of genes of a functional class in each cluster, by the total number of genes in that functional class. For a representation of all twenty-six functional classes in all ten clusters see Additional File 1 (Figure. S2).

The genes within each cluster were analysed and the percentage of twenty-six functional classes  in each cluster was determined [see Additional file 1 – Supplementary Figure. S2]. Figure 3 shows the distribution of ten functional classes throughout the clusters.

Of the functional classes, 'hypothetical, unclassified, unknown' was the most abundant, comprising at least 25% of the total number of genes in each cluster. As expected, clusters 4 and 5 (up-regulation in LP planktonic culture and developing biofilms) contain many genes in functional classes associated with growth (ie. 'transcription, RNA processing and degradation'; 'translation, post-translational modification, degradation', 'nucleotide biosynthesis and metabolism', 'DNA replication, recombination, modification and repair', 'cell division', 'amino acid biosynthesis and metabolism') (Figure 3). There is also a large percentage of genes from the classes 'transcription, RNA processing and degradation', 'cell wall/LPS/capsule' and 'cell division' in clusters 7 (up-regulation in LP planktonic culture) and 9 (up-regulation in LP and SP planktonic culture) (Figure 3). The 'motility and attachment' class features most prominently in clusters 6 (up-regulation in SP planktonic) and 8 (up-regulation in developing biofilms) (Figure 3); many genes in cluster 6 are involved in the biosynthesis and assembly of flagellar (essential for swimming and swarming motility) whereas cluster 8, contains many genes involved in biosynthesis and assembly of type-IV pili (essential for twitching motility). The regulators of biosynthesis of these motility structures are also present in these groups. Perhaps, the most interesting results are the appearance of a large percentage of genes involved in 'chemotaxis' and genes from the functional class 'phage, transposon, or plasmid' in clusters 1 (up-regulation in the confluent biofilm time points – expression peak 14 hrs) and 5 (up-regulation in LP planktonic culture and developing biofilms), respectively (discussed later) (Figure 3).

Gene lists for each cluster were compiled, together with their microarray expression results [see Additional file 3, Tables S3 – S12]. The following section describes some of the key genes found in the different expression profiles.

### Genes up-regulated in confluent-biofilms, and genes up-regulated in confluent-biofilms and in SP planktonic culture

Cluster 1 and cluster 2 both contain genes up-regulated in the confluent biofilm time points with peak expression occurring at 14 hrs and 48 hrs, respectively [see Additional file 3, Supplementary Tables S3 and S4]. Cluster 3 contains genes up-regulated in SP planktonic culture as well as the confluent biofilm time points [see Additional file 3, Supplementary Table S5].

**Figure 4:**
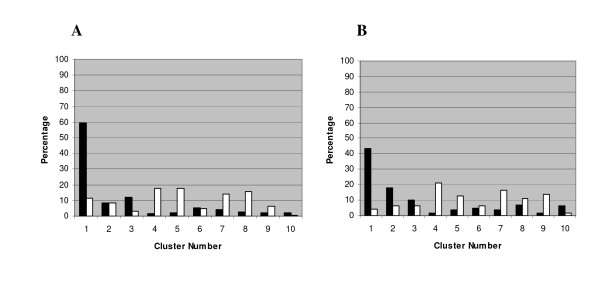
**Percentage of a) RpoS and b) QS regulated genes in each cluster**. a) Percentage obtained by dividing the number of RpoS activated or RpoS repressed genes in each cluster by the total number of RpoS activated or RpoS repressed genes. b) Percentage obtained by dividing the number of QS activated or QS repressed genes in each cluster by the total number of QS activated or QS repressed genes. Black columns – activated genes; white columns, repressed genes. QS and RpoS regulated genes were identified under planktonic growth conditions [11-13].

Although the previously identified QS and RpoS regulated genes were identified under planktonic growth conditions [11-13], it is known that these regulatory systems play an important role in biofilm maturation [14-16]. Therefore, we utilized the available QS and RpoS transcriptomic datasets [11-13] to determine the distribution of these genes throughout the 10 clusters. The majority of genes previously found to be regulated by QS and RpoS in planktonic culture were also expressed in confluent biofilms. 423 of the 504 genes found to be up-regulated by RpoS [12] were in the gene list used for the cluster analysis and 80.1% of these genes were found distributed between clusters 1, 2 and 3 (Figure 4a). 434 of the 534 genes (in total) shown to be up-regulated in two QS studies [11,13] were in the gene list used for the cluster analysis and 71% of these genes were found distributed amongst clusters 1, 2 and 3 (Figure 4b). This percentage would probably have been higher had full strength LB been used rather than the reduced strength LB broth (20%) used to culture planktonic and biofilm cells in this study. Taking into account the very different conditions we used when compared to previous QS and RpoS transcriptomic studies [11-13] and also considering the cell densities of the different growth states we used, the distribution of the QS and RpoS regulated genes over the 10 clusters that we obtained would be expected.

As many *P. aeruginosa *virulence determinants are regulated by QS, it is not surprising that the following are found in clusters 1, 2 and 3; elastase precursor (*lasB*, cluster 1), phenazine biosynthetic genes (PA1901 – PA1905, PA4209, PA4210-PA4211 cluster 1), and rhamnosyltransferases involved in rhamnolipid biosurfactant synthesis (*rhlAB *cluster 3, *rhlC *cluster 2). Many of the genes in clusters 1, 2 and 3 are activated in response to stress in *P. aeruginosa *or are orthologues of stress induced genes in other bacteria; general stress (PA1753, PA2190, *cspD*, *rpoS*, and *rmf*), nutrient stress (*glgA*, *glgB*, *glgP *– discussed in the next section), oxidative stress (*katE *– discussed in the next section), anaerobicity (*anr*), and osmolarity (*osmC*, *osmE *and *osmY*). Cluster 2 contains the gene encoding the transcriptional regulator – Anr, the anaerobic regulator of the arginine deiminase (ADI) and nitrate reductase pathways. Anr induces the ADI pathway in *P. aeruginosa *during oxygen limitation [17] enabling this organism to use arginine as an energy source. Three of the four genes of the ADI pathway (*arcD*, *arcB*, *arcC*) are present in clusters 1 and 3. Interestingly, cluster 1 includes a six ORF cluster *napEFDABC *(PA1172 – PA1177) and PA4130 which are thought to be involved in nitrate reduction, and nitrite or sulfite reduction, respectively. *P. aeruginosa *is known to use nitrate or nitrite as a terminal electron acceptor under anaerobic conditions [18]. Biofilms are known to be heterogeneous environments and it is known that even if biofilms are cultured under aerobic conditions there is little oxygen deep within mature biofilms [19] and in the CF lung [20]. Therefore, we can speculate that in our study cells at the surface of the biofilm are in an aerobic environment whereas cells nearest the filter surface after 48 hours biofilm growth are in a localised anaerobic environment. It is therefore not surprising that the gene encoding the anaerobic regulator Anr was found in cluster 2 with expression peaking after 48 hours biofilm growth.

Cluster 1 contains PA1753, a homologue of an *E. coli *universal stress protein (UspA). UspA is thought to have a general protective function, is one of most abundant proteins in growth arrested cells and is stimulated by numerous conditions such as SP conditions, nutrient limitation, and exposure to oxidants and antibiotics [21]. Cluster 2 contains PA2622 which is 71% similar to the *cspD *gene product of *Escherichia coli*, a member of the CspA cold shock stress adaptation family of proteins [22]. Although *E. coli *CspD is not induced by cold shock it is dramatically induced by SP growth and glucose starvation [22].

Other general stress genes in cluster 2 are *rpoS*, PA2190 (discussed in the next section) and *rmf*. *rmf *encodes a putative protein which is 66% similar to the characterised ribosome modulation factor of *E. coli *and was very highly expressed in the confluent biofilm time points. In *E. coli *this protein has been shown to be expressed during the transition from LP to SP growth and in slow growing cells and its role is to associate with 70S ribosomes, converting them into a dimeric form and reducing protein synthesis [23,24].

Cluster 1 contains PA0059, PA4876 and PA4739, homologues of three *E. coli *proteins (*osmC*, *osmE*, *osmY*) that are osmotically induced. Interestingly, in *E. coli *it has been previously found that bacteria within biofilms encounter higher osmolarity conditions than bacteria in a planktonic state [25].

In cluster 2 are two genes that encode lectins, *lecA *(PA2570 – galactophilic lectin) and *lecB *(fucose-binding lectin) which are very highly expressed in the three confluent biofilm time points (>2.5 fold up-regulated, when compared to the three other conditions, *P *< 0.05). Interestingly, a *lecB *deficient mutant has previously been found to be significantly impaired in biofilm formation [26].

Three genes of the *mexGHI-opmD *efflux system were found in cluster 2. This efflux pump is thought to be involved in acyl-homoserine lactone (AHL) homeostatis and resistance to vanadium, but mutants defective in this efflux system do not display decreased antibiotic resistance [27].

*P. aeruginosa *has five (I to V) 'clusters' (which will be called 'sets' to avoid confusion) of chemotaxis genes. Genes from sets I, II and III are found in cluster 1 and genes from set V is found in cluster 3. Why genes from these chemotaxis sets are up-regulated under biofilm conditions in which motility biosynthetic genes (flagellar and type IV pili) are down-regulated is not clear. One explanation could be that these chemotactic sets are involved in environmental sensing and responding to stress under biofilm conditions [12].

### A genome region associated with stationary phase growth (planktonic and biofilm)

**Figure 5:**
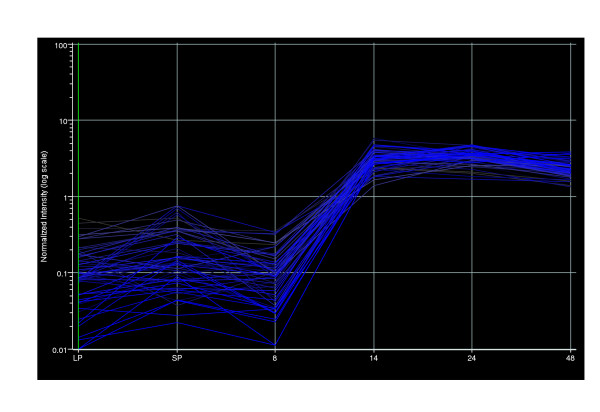
**Gene expression profile of genome region PA2134-PA2190**. X-axis labels – LP, LP planktonic culture; SP, SP planktonic culture; 8, 14, 24 & 48, biofilm time points.

Whilst studying the genes in clusters 1 and 2 it became apparent that there was a region of the genome (PA2134 – PA2190; 60.69 Kbp from position 2349488 to 2410181) that had the same expression profile; up-regulation in the confluent biofilm time points (14, 24 and 48 hrs) (Figure 5).

32 of these 57 sequential genes have previously been found to be QS and/or RpoS regulated [11-13] using SP planktonic cultures of higher cell density than that of the SP planktonic cultures used in this study. Therefore, it is possible that this region could be associated with stationary phase growth (SP planktonic culture and/or confluent biofilms). There is no putative function assigned to the majority (40) of these genes. Interestingly this region contains genes with possible roles in the accumulation and breakdown of storage materials (glycogen, trehalose), oxidative stress protection (two catalases; *katE, katN*) and general stress (PA2190) (Table 2). *glgA*, *glgB*, *glgP *encode enzymes with putative roles in glycogen synthesis and degradation (GlgA – a probable glycogen synthase, GlgB – a 1, 4-alpha-glucan branching enzyme and GlgP – a putative glycogen phosphorylase). An *E. coli glgA *mutant was impaired in biofilm formation [28] and GlgP has been suggested to have a role in the slow degradation of endogenous glycogen during long stationary conditions [29]. This region also contains three probable glycosyl hydrolases (PA2160, PA2162, PA2164) which may also be involved in the hydrolysis of storage polysaccharides and PA2190 which is 62% similar to the glucose or phosphate starvation inducible *gsiB *general stress gene of *Bacillus subtilis *[30]. The two catalase genes present in this region; *katE *which encodes catalase HPII and *katN *which encodes a non-haem catalase, could aid the major housekeeping catalase KatA (PA4236) in SP conditions and in particular in the protection of biofilms against hydrogen peroxide.

Given that amongst this region there are genes that appear to be involved in general stress, glycogen accumulation and breakdown, and oxidative stress survival, it would be interesting to determine whether there are any genomic or expression differences in this region between different isolates (clinical and environmental) and if so, whether there is any difference in survival of these isolates under long term stationary conditions (biofilm and planktonic).

### Genes up-regulated in LP planktonic culture and 8 hrs developing biofilms

Clusters 4 and 5 both contain genes up-regulated in LP planktonic culture and in developing biofilms (8 hrs), with expression peaking in LP planktonic culture and developing biofilms, respectively [see Additional file 3, Supplementary Tables S6 and S7]. Genes involved in energy generation are well represented in clusters 4 and 5, for example: ATP synthase genes (PA5553 – PA5561, cluster 4), NADH dehydrogenase genes (PA2638 – PA2649, clusters 4 and 5), genes encoding enzymes involved the tri-carboxylic acid (TCA) cycle – succinate dehydrogenase, 2-oxoglutarate dehydrogenase, succinyl-CoA synthetase (PA1581 – PA1589, cluster 4). Also, present in cluster 5, are the chaperonins (*groEL *and *groES*) and a well characterised transcriptional regulator – Vfr, which regulates the *las *quorum sensing system [31] and twitching motility [32]. Many genes involved in protein secretion/export were present in clusters 4 and 5, for example; genes encoding the primary sec-dependent protein translocation pathway (*secA*, *secB*, – cluster 5; *secF*, *secD*, *secY*, *secG *– cluster 4), *tatB *(cluster 5)/*tatC *(cluster 4) of the twin arginine translocation system (Tat) and signal peptidase I encoded by *lepB *(cluster 4) which cleaves signal peptides off proteins translocated across biological membranes. Genes involved in lipopolysaccharide biosynthesis (PA3145 – PA3158) are found in cluster 4.

A previous *P. aeruginosa *strain PAO1 microarray study suggested phage induction to be important for gene transfer in biofilms, as a region of genes (PA0718 – PA0727) of the functional class 'phage, transposon, or plasmid' were found to be highly up-regulated in mature biofilms when compared to planktonic culture [15]. Therefore, it would be expected that these genes would appear in clusters 1 and 2 (ie. the late biofilm time points). However, only one gene from this region was found in cluster 1 (PA0723). In contrast, we found cluster 5 to contain the highest percentage of 'phage, transposon, or plasmid' functional class genes, all of which were in a different region (PA0616-PA0624, PA0632-PA0636, PA0643-PA0646) to the previous study. Many of these genes have homology to *P. aeruginosa *R pyocin-related phage family genes (e.g. phi CTX, PS17). Phi CTX is a temperate phage that produces a pore-forming cytotoxin [33] and R pyocin is a type of bacteriocin that has a structure similar to contractile phage tails [34,35]. PA0622 and PA0623 encode proteins with high homology (91 and 84%, respectively) to contractile tail proteins of phage PS17. Therefore, it is possible that these phage genes which have recently been shown to be induced in response to hydrogen peroxide stress [36] may play a role in virulence under actively growing conditions (LP planktonic culture and 8 hr biofilm) which could be encountered *in vivo*.

### Genes up-regulated in SP planktonic culture

Cluster 6 contains genes up-regulated in SP planktonic culture [see Additional file 3, Supplementary Table S8]. As discussed above this cluster is unique, as many of the genes displaying this expression profile are involved in the biosynthesis, assembly and regulation of flagellar motility.

Other genes that display this profile suggest magnesium (*phoQ*, *oprH*, *mgtA*, *mgtE*) and potassium (*KdpFABCD*) may be limiting in the SP planktonic culture conditions used in this study. Genes known to be stimulated in response to magnesium starvation are; the *oprH-phoPQ *operon which encodes an outer-membrane protein (OprH) and a two component regulatory system (PhoP/Q) [37], *mgtA *(a ATP-dependent Mg^2+ ^transporter homologue) [38], and *mgtE *(a probable Mg transporter). Whilst the *kdp *gene cluster (PA1632 – 1637) encodes a putative high affinity K^+ ^ion transport system. However, the expression profile of these genes differs greatly to the *kdpABC *operon of *Staphylococcus aureus*, which has been found to be up-regulated in biofilms compared to planktonic culture [39].

In this study the general stress gene *recA *was found to be up-regulated in SP planktonic culture, unlike in *E. coli *in which *recA *has been found to be up-regulated in biofilms [40]. This cluster also contains *algU *the *P. aeruginosa *orthologue of *E. coli *extreme stress sigma factor σ^E^.

### Genes up-regulated in planktonic culture; LP, or LP and SP planktonic culture

Cluster 7 contains genes up-regulated in LP planktonic culture whereas cluster 9 contains genes up-regulated in LP and SP planktonic culture [see Additional file 3, Supplementary Tables S9 and S11]. These two clusters contain genes encoding two characterised multidrug efflux systems (MexAB-OprM, and MexXY) between them. Genes from two putative multidrug efflux systems (PA2527, PA2528, PA1541) were also present in these two clusters together with a two-component regulatory system (PmrA-PmrB) that regulates resistance to polymyxin B and cationic antimicrobial peptides. This two-component system is involved in the regulation of a putative LPS modification operon (PA3552 – PA3559) [41] which is also present in cluster 7. Biofilms are well known for their decreased susceptibility to antibiotics when compared to planktonic cells. However, no previously characterised antibiotic efflux system was found to be up-regulated in the static biofilm system used in this study. It is well known that other factors such as slow bacterial growth, oxygen limitation and decreased penetration of antibiotic through the exopolysaccharide matrix play a role in the decreased susceptibility of biofilms to antibiotic therapy [42,43]. However, recent studies have shown biofilm specific induction of the MexCD-OprJ efflux system in the presence of azithromycin [44] and biofilm specific induction of genes from two probable efflux systems upon exposure to tobramycin [15].

### Genes up-regulated in 8 hrs developing biofilms

Cluster 8 contains genes up-regulated in developing biofilms (8 hrs) [see Additional file 3, Supplementary Table S10]. Given the importance of type-IV pili in microcolony formation [45] it is not surprising that many of the genes displaying this expression profile are involved in the biosynthesis, assembly and regulation of type-IV pili.

Although it is expressed highly under all conditions the transcriptional regulator *mvaT *was also included in this cluster as expression peaked in 8 hr biofilms. MvaT is a global regulator of gene expression [46] and negatively regulates the *cupA *gene cluster (chaperone-usher pathway) which has previously been found to be required for biofilm formation on abiotic surfaces [47,48] but is not expressed in our study. Other genes that display the cluster 8 profile are a chitin binding protein precursor (*cbpD*), bacterioferritin comigratory protein (*bcp*), a putative cold shock protein (PA1159) and the ecotin precursor which is thought to inhibit neutrophil elastase [49] and could play a protective role against the host immune system in developing biofilms.

### Genes up-regulated in SP planktonic culture and in developing biofilms (8 hrs)

Three functional classes well represented in cluster 10 are those of amino acid biosynthesis and metabolism, fatty acid metabolism and carbon compound catabolism [see Additional file 3, Supplementary, Table S12]. Three gene clusters that illustrate this are; *bkdA1A2B-lpdV*, *gnyDBHAL *and *soxBDAG*. The *bkd *operon encodes a multi-enzyme complex branched-chain keto acid dehydrogenase involved in metabolism of valine, leucine and isoleucine [50]. Whereas the *gny *gene cluster encodes enzymes involved in degradation of acyclic isoprenoids [51] and the *sox *gene cluster encodes a putative sarcosine oxidase which catalyses the oxidative demethylation of sarcosine (*N*-methylglycine) to glycine, formaldehyde and hydrogen peroxide.

## Conclusion

The three clustering methods employed in this study (K-means, K-means spectral and hierarchical clustering) allowed us to sort this large transcriptomic dataset into genes with similar expression profiles. This enabled direct visualisation of gene expression common to two or more culture conditions and unique to single conditions. Confidence in our results was gained from the fact that for equivalent settings of the parameters all three clustering methods gave broadly the same grouping of expression profiles. Thus, the distinct expression clusters obtained together with the genes with low expression and consistent expression [see Additional file 2, Table S1 and S2] over all conditions, gives a complete picture of gene expression for this organism under conditions relevant to its lifestyle.

Here we have discussed a selection of genes representative of each cluster but given the large number of hypothetical genes and un-characterised orthologues in each cluster, there is still much to understand about the molecular and genetic basis of planktonic and biofilm growth of *Pseudomonas aeruginosa*. However, we believe that the availability of this dataset as a resource for the research community will accelerate the determination of the roles of these genes.

Structuring this data in clusters provided a clear illustration of the main expression profiles of many novel genes, such as genes from the functional classes 'chemotaxis' and 'phage, transposon, or plasmid' which are found in clusters 1 and 5 respectively. The cluster analysis also enabled us to observe the up-regulation of many individual genes in confluent biofilms, for example; 1) un-characterised orthologues associated with general stress (PA1753, PA2190, *cspD*, *rmf*), nutrient stress (*glgA*, *glgB*, *glgP*), oxidative stress (*katE*), and osmolarity (*osmC*, *osmE *and *osmY*), 2) the characterised anaerobic transcriptional regulator Anr (encoded by *anr*). It also facilitated the identification of a region of 57 sequential genes (PA2134 – PA2190) which demonstrate the same expression profile (up-regulation in the confluent biofilm time points). 32 of these genes have previously been found to be QS and/or RpoS regulated [11-13].

Given the rise in antibiotic resistance, its ability to survive in the less antibiotic susceptible state – as a biofilm and the natural recalcitrance of this important human pathogen to many commonly used antibiotics, this dataset should aid in the pursuit of novel therapies against *P. aeruginosa*. Novel QS inhibitors have already been used with some success *in vitro *[52,53], and the increasing sophistication of proteomic and transcriptomic technology will accelerate the identification of other novel targets. For example this dataset could aid in the selection of new protein targets consistently expressed under all conditions (biofilm and planktonic) [see Additional file 2, Supplementary Table S2] or against protein targets expressed exclusively in the biofilm state. However, as biofilms are heterogeneous communities of cells, care should be taken when selecting biofilm specific targets and the spatial expression of these individual targets within biofilms should be studied. Other authors have found that different biofilm structures are formed when different media is used (uniform densely packed biofilms and mushroom shaped microcolonies) [54], which indicates that there is also a need to establish the difference in gene expression profiles between these different biofilm structures.

## Methods

### Bacterial strains

*Pseudomonas aeruginosa *wild-type strain PAO1 [55] was used for both biofilm and planktonic culture expression analysis.

### Biofilm modelling, confocal imaging, planktonic phase culturing, RNA isolation, cDNA labelling and microarray scanning

Biofilm modelling, confocal imaging, planktonic phase culturing, RNA isolation, cDNA labelling and microarray scanning were performed as previously described [5]. The RNA samples used in this analysis were biological replicates, as they were derived from three independent cultures for each condition.

### Quantitative reverse transcriptase PCR (qRT-PCR)

Planktonic and biofilm gene expression profiles generated using Affymetrix GeneChip arrays were verified using qRT-PCR. The same RNA samples used for the array analysis were also used for the qRT-PCR. 500 ng of RNA was reverse transcribed using the iScript cDNA Synthesis Kit as described by the manufacturer (BioRad Laboratories) and as a control the reaction was performed without reverse transcriptase (RT). Spectrophotometry was used to quantitate total cDNA and 67.5 ng of cDNA was used for each reaction using universal master mix (Applied Biosystems). Gene specific primers and probes were designed using Primer Express Software (Applied Biosystems), the sequences of which are shown in Table 3. Probes (purchased from Sigma-Genosys) were labeled 5' with 6-carboxyfluorescein (FAM) as the reporter and 3' with 6-carboxytetramethylrhodamine as the quencher (TAMRA). Each reaction was carried out in triplicate (in the presence of a no template control) using a model ABI 7900 HT thermal cycler (Applied Biosystems); thermal cycling parameters were 2 minutes at 50°C, 10 minutes at 95°C, and 40 cycles of 95°C for 15 seconds and 60°C for 1 minute. Calculation of expression value's for each gene (where cycle threshold is abbreviated as Ct): Ct (+RT) - Ct (-RT) = cCt (corrected CT). ΔCt = cCt (sample gene) - cCt (control gene, *clpP*). Expression value = 2^-ΔCt^. *clpP *was used as a control as it was found to be consistently expressed under all six conditions using microarray profiling [see Additional file 2, Supplementary Table S2].

### Cluster analysis of microarray data

Before data processing, all microarray data was globally scaled to set the average signal intensity of each array to a target signal of 100.

We began the transcriptome analysis by filtering out 1429 genes showing very low expression for all six different conditions (under 50 signal units for all three replicates). A 1-way ANOVA (assuming unequal variances) was then performed which removed a further 805 genes that did not vary significantly between different conditions [see Additional file 2, Supplementary Table S2]. For the remaining 3315 genes, we averaged the values of the three replicates for each of the six conditions, and then normalized these averaged profiles to zero mean and unit variance. We used the correlation coefficient as distance measure between gene profiles, and we clustered them using K-means clustering [8], K-means spectral clustering [9,10] and hierarchical clustering [8]. The first two methods were applied using a value of K (i.e. number of clusters) of 4, 7, 8, 9, 10, 12, 13, 15, 16 and 19. For the hierarchical clustering we tried the complete, centroid and ward linkage metrics for building the hierarchical tree and different values of the threshold for cutting it. An important finding is that for equivalent settings of the parameters of these different algorithms we obtained broadly the same groupings of genes. This revealed the existence of distinct expression profiles in the transcriptomic data from these six conditions. As within the paper we discuss the results obtained using K-means clustering with K = 10, in the following section we discuss some details of K-means clustering. K-means is a standard algorithm for partitioning *N *data points into *K *disjoint subsets *S*_*j *_containing *N*_*j *_data points. The algorithm consists of a re-estimation procedure that attempts to minimize the sum-of-squares criterion:

J=∑j=1κ∑n∈Sj|xn−μj|2,     (A)
 MathType@MTEF@5@5@+=feaafiart1ev1aaatCvAUfKttLearuWrP9MDH5MBPbIqV92AaeXatLxBI9gBaebbnrfifHhDYfgasaacH8akY=wiFfYdH8Gipec8Eeeu0xXdbba9frFj0=OqFfea0dXdd9vqai=hGuQ8kuc9pgc9s8qqaq=dirpe0xb9q8qiLsFr0=vr0=vr0dc8meaabaqaciaacaGaaeqabaqabeGadaaakeaacqWGkbGscqGH9aqpdaaeWbqaamaaqafabaGaeiiFaWNaemiEaG3aaSbaaSqaaiabd6gaUbqabaGccqGHsisliiGacqWF8oqBdaWgaaWcbaGaemOAaOgabeaakiabcYha8naaCaaaleqabaGaeGOmaidaaaqaaiabd6gaUjabgIGiolabdofatnaaBaaameaacqWGQbGAaeqaaaWcbeqdcqGHris5aaWcbaGaemOAaOMaeyypa0JaeGymaedabaGae8NUdSganiabggHiLdGccqGGSaalcaWLjaGaaCzcamaabmaabaGaeeyqaeeacaGLOaGaayzkaaaaaa@4E06@

where *x*_*n *_is a vector representing the *n*th data point and *μ*_*j *_is the mean of the data points in *S*_*j*_.

When using this algorithm for clustering gene expression profiles, each profile was considered a data point, and the distance in (A) was the correlation coefficient between *x*_*n *_and *μ*_*j*_. This amounts to say that we are learning the expression profiles which are the centers of the clusters (the thick yellow line Figure 1b).

The clearest representation of the expression profiles within the dataset was obtained with a K = 10 (Figure 1a and 1b). The 10 clusters are defined in Table 1. The clusters found were quite stable with respect to different random initialization of the algorithm. Gene lists for each cluster were compiled, together with their microarray expression results [see Additional file 3, Tables S3 – S12]. We also performed the analysis directly on the experimental data, without averaging the replicates. As expected, the results obtained [see Additional file 1, Supplementary Figure S3] were similar to the ones presented in Figure 1 although more noisy.

## Authors' contributions

RDW performed biofilm and planktonic culturing, microarray experiments, quantitative RT-PCR, data analysis and drafted the manuscript. AlP performed the cluster analysis and drafted the manuscript. JH performed data analysis. AnP and MS supervised the study and reviewed the manuscript. EL initiated the study. MC initiated and supervised the study, and reviewed the manuscript.

## Supplementary Material

Additional file 1 **Figure S1. Results of clustering the expression data with K-means spectral clustering (K = 10) and hierarchical clustering with ward linkage**. Shows the expression profiles obtained through these two methods of clustering. It also compares these two clustering methods with K-means clustering (K = 10) which is shown in the paper. For this analysis the average of replicates for each condition was used. **Figure S2. Functional class representation in the ten clusters created by K-means clustering**. From this figure it can be determined what percentage of genes in each of twenty-six functional classes are present in each cluster. **Figure S3. Results of K-means clustering (K = 10) of expression data from all replicates from all six conditions**. Shows an Eisen diagram of the expression profiles grouped according to the results of the clustering and a graphical plot of the profiles in each cluster. Click here for file

Additional file 2 **Table S1. Contains a list of all genes that display low expression in all six conditions studied**. This list contains genes showing very low expression (under 50 signal units for all three replicates) for all six different conditions. These 1429 genes were not used in the clustering analysis. The table displays the average microarray expression results for each gene for each condition. **Table S2. Contains a list of genes in which gene expression did not vary significantly between the six different conditions**. This list was obtained using a 1-way ANOVA (assuming unequal variances) and genes in this list were not used in the clustering analysis. The table displays the average microarray expression results for each gene for each condition. Click here for file

Additional file 3 **Tables S3 – S12. Genes present in the clusters obtained by K-means clustering (K = 10)**. The genes in clusters 1 -10 are displayed together with their average microarray expression results for each condition. Click here for file

## Figures and Tables

**Table 1:** Description of expression profiles obtained by K-means clustering (K = 10) of microarray data

**Cluster No**	**Up-regulation in:**
1	confluent biofilm time points (expression peak – 14 hrs)
2	confluent biofilm time points (expression peak – 48 hrs)
3	confluent biofilm time points and in SP planktonic culture
4	LP planktonic culture (expression peak) and in developing biofilms (8 hrs)
5	LP planktonic culture and in developing biofilms (8 hrs) (expression peak)
6	SP planktonic culture
7	LP planktonic culture,
8	developing biofilms (8 hrs)
9	LP and SP planktonic culture
10	SP planktonic culture and in developing biofilms (8 hrs)

**Table 2:** Genes present in region PA2134 – PA2190 with an assigned putative function^a^

**ORF^b^**	**Product Name**	**Gene Name**
PA2135	probable transporter	
PA2138	probable ATP-dependent DNA ligase	
PA2140	Probable metallothionein	
PA2142	probable short-chain dehydrogenase	
PA2144	glycogen phosphorylase	*glgP*
PA2147	catalase HPII	*katE*
PA2152	probable trehalose synthase	
PA2153	1, 4-alpha-glucan branching enzyme	*glgB*
PA2155	probable phospholipase	
PA2158	probable alcohol dehydrogenase (Zn-dependent)	
PA2160	probable glycosyl hydrolase	*glgX*
PA2162	probable glycosyl hydrolase	
PA2164	probable glycosyl hydrolase	
PA2165	probable glycogen synthase	*glgA*
PA2177	Probable sensor/response regulator hybrid	
PA2185	non-heme catalase KatN	*katN*
PA2188	probable alcohol dehydrogenase (Zn-dependent)	

^a ^Genes are identified by ORF designation, gene name and product name

^b ^All other genes in this region are of the class 'hypothetical, unclassified, unknown'.

**Table 3:** Sequences of primers and TaqMan probes used for qRT-PCR

**Primer or probe^a^**	**Oligonucleotide sequence 5'→3'**
FliE F	GGAAATGCGTTCCATGCAA
FliE R	GGAGAGCATTTCCGAAAAGCT
FliE P	TTCAGGCGCCGGCCGAAG
PA0020 F	GCCGGCCTATGGCATCTT
PA0020 R	GCGTTGATGCCGAGGACTT
PA0020 P	CCAGGGCAAGGTCTACATCGATCCG
ClpP F	GGTGGTTGCTCAGTTGCTGTT
ClpP R	CGGCGAGTTGATGTAGAGATGA
ClpP P	CTGGAGGCTGAAAATCCCGAGAAGGA
PA2184 F	GCGCGGCTGGAACACTAT
PA2184 R	CCATGGGTTTCCTCGATGTG
PA2184 P	CGCAACTGAAGACGCGGATCGA
PA5555 F	CGCTGGTGGCCGATGA
PA5555 R	GGCGTCGGGTTCGTAGAGA
PA5555 P	TCAGGAGCTGAAGCACCACTGGGACT

**a**. Forward primers (F), reverse primers (R) and TaqMan probes (P) are displayed.
