# Supplementary material for: Clustering of Pseudomonas aeruginosa transcriptomes from planktonic cultures, developing and mature biofilms reveals distinct expression profiles
Source: BMC Genomics. 2006 Jun 26;7:162. doi: 10.1186/1471-2164-7-162 (PMC1525188; doi:10.1186/1471-2164-7-162)
Supplement: Additional file 1 — Figure S1. Results of clustering the expression data with K-means spectral clustering (K = 10) and hierarchical clustering with ward linkage. Shows the expression profiles obtained through these two methods of clustering. It also compares these two clustering methods with K-means clustering (K = 10) which is shown in the paper. For this analysis the average of replicates for each condition was used. Figure S2. Functional class representation in the ten clusters created by K-means clustering. From this figure it can be determined what percentage of genes in each of twenty-six functional classes are present in each cluster. Figure S3. Results of K-means clustering (K = 10) of expression data from all replicates from all six conditions. Shows an Eisen diagram of the expression profiles grouped according to the results of the clustering and a graphical plot of the profiles in each cluster. [file 1471-2164-7-162-S1.doc]

**Additional figures**

**I K-means spectral clustering (K=10) and hierarchical clustering**

**A) B)**

Cluster

No

1

2

3

4

5

6

7

8

9

10


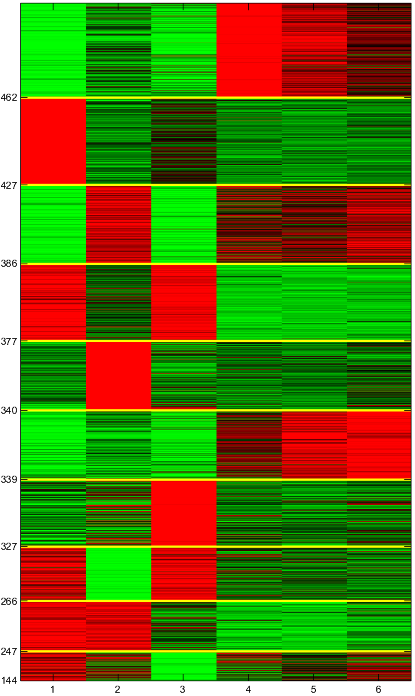

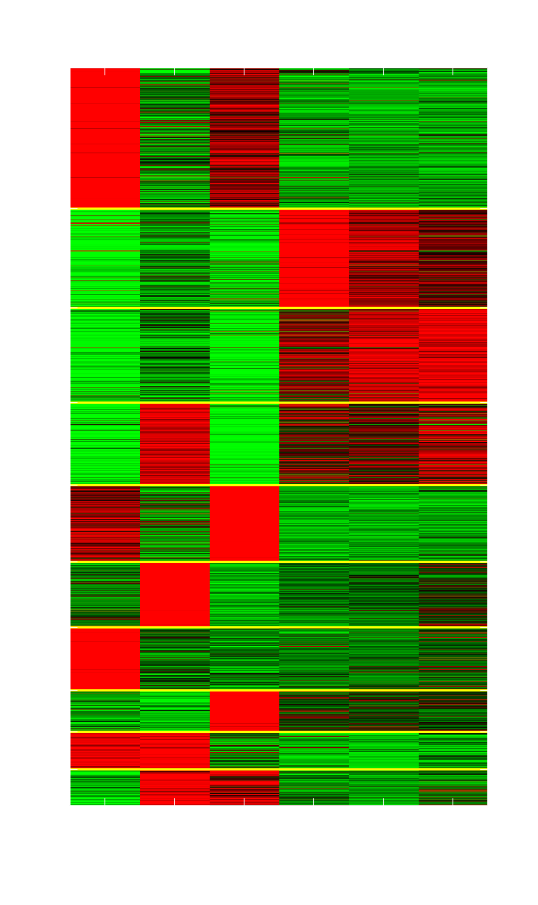


633-

446-

426-

370-

345-

294-

283-

187-

168-

163-

Cluster

No

1

2

3

4

5

6

7

8

9

10

LP SP 8 14 24 48

LP SP 8 14 24 48

**Figure S1. Clustering of expression data.** The three replicates for each of the six conditions were averaged, and then normalized to zero mean and unit variance. The Eisen diagrams show expression profiles grouped according to the results of the clustering. Red denotes that the value observed is above the mean of the observation across the dataset. The similarity between these two figures and figure 1 can be visually appreciated. A) Eisen diagram showing the results of hierarchical clustering (ward linkage). Notice how clusters 1, 2, 3, 4, 5, 6, 7, 8, 9 and 10 in Fig S1 A) are similar to clusters 4, 1, 2, 3, 5, 6, 7, 8, 9 and 10 in Figure 1. **Note:** the figure is an eisen diagram of the clusters obtained by cutting the hierarchical tree. B) Eisen diagram showing the results of K-means spectral clustering (K=10). Notice how clusters 1, 2, 3, 4, 5, 6, 7, 8, and 9 in Fig S1 B are similar to clusters 1, 7, 3, 4 or 5, 6, 2, 8, 4 or 5 and 9 in Figure 1. The y-axis shows the number of genes in each cluster. The labels on the X-axis denote the different expression conditions: LP**,** LP planktonic culture; SP, SP planktonic culture; 8, 14, 24 and 48, biofilm time points.

**II Functional class representation of the ten different clusters created by K-means clustering.**

**Figure. S2.** Functional class representation of the ten different clusters created by K-means clustering. All twenty-six functional classes are shown. Percentages were obtained by dividing the number of genes of a functional class in each cluster, by the total number of genes in that functional class. X-axis – functional class number (see key below), Y-axis – percentage.

| **Functional class** | **Number** |
| --- | --- |
| Adaptation, protection | 1 |
| Amino acid biosynthesis and metabolism | 2 |
| Antibiotic resistance and susceptibility | 3 |
| Biosynthesis of cofactors, prosthetic groups and carriers | 4 |
| Carbon compound catabolism | 5 |
| Cell division | 6 |
| Cell wall / LPS / capsule | 7 |
| Central intermediary metabolism | 8 |
| Chaperones & heat shock proteins | 9 |
| Chemotaxis | 10 |
| DNA replication, recombination, modification and repair | 11 |
| Energy metabolism | 12 |
| Fatty acid and phospholipid metabolism | 13 |
| Hypothetical, unclassified, unknown | 14 |
| Membrane proteins | 15 |
| Motility & Attachment | 16 |
| Nucleotide biosynthesis and metabolism | 17 |
| Protein secretion/export apparatus | 18 |
| Putative enzymes | 19 |
| Related to phage, transposon, or plasmid | 20 |
| Secreted Factors (toxins, enzymes, alginate) | 21 |
| Transcription, RNA processing and degradation | 22 |
| Transcriptional regulators | 23 |
| Translation, post-translational modification, degradation | 24 |
| Transport of small molecules | 25 |
| Two-component regulatory systems | 26 |

**III K-means clustering with K=10 of microarray data from all replicates from all six conditions**

In addition to the K-means clustering with K=10 analysis described in the manuscript, we also performed K-means clustering with K=10 directly on the experimental data, without first averaging the replicates. As expected, the results obtained (presented below) are similar to those in Figure 1, although more noisy (this is generally reported in the literature, and it justifies taking the means of replicates to denoise gene expression data). It can be visually appreciated that there is a correspondence between the clusters shown here and the clusters in figure 1 (clusters 1, 2, 3, 4, 5, 6, 7, 8, 9 and 10 here correspond to clusters 4, 6, 7, 1, 2, 5, 3, 9, 8 and 10 in Figure 1).

**A)** **B)**

Cluster No


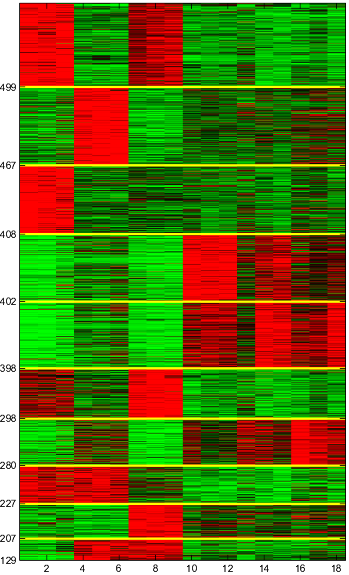

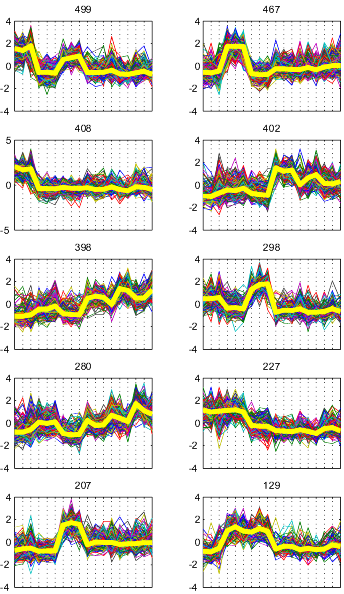


1

2

3

4

5

6

7

8

9

10

LP SP 8 14 24 48

**Figure S3. Results of K-means clustering (K=10) of microarray data from all replicates from all six conditions.** A) Eisen diagram of the profiles grouped according to the results of the clustering. Red denotes that the value observed is above the mean of the observation across the dataset. The y-axis shows the number of genes in each cluster. The labels on the X-axis denote the 3 replicates from each of the different expression conditions: LP**,** LP planktonic culture; SP, SP planktonic culture; 8, 14, 24 and 48, biofilm time points. B) Plot of the profiles in each cluster. The X-axis shows the conditions in the same order as A). The thick yellow lines represent the learned cluster centres.
